# IgG Antibodies Develop to Spike but Not to the Nucleocapsid Viral Protein in Many Asymptomatic and Light COVID-19 Cases

**DOI:** 10.3390/v13101945

**Published:** 2021-09-28

**Authors:** Maria Tutukina, Anna Kaznadzey, Maria Kireeva, Ilya Mazo

**Affiliations:** 1 Skolkovo Institute of Science and Technology, Bolshoy Boulevard 30, bld. 1, 121205 Moscow, Russia; masha306@gmail.com; 2 Institute of Cell Biophysics, Russian Academy of Sciences, Federal Research Centre “Pushchino Scientific Centre of Biological Research of Russian Academy of Sciences”, Institutskaya 3, 142290 Pushchino, Russia; 3 Institute for Information Transmission Problems, Russian Academy of Sciences, Bolshoy Karetny per. 19, 127051 Moscow, Russia; 4 VirIntel, LLC, Gaithersburg, MD 20877, USA; maria.kireeva@virintel.com (M.K.); ilya.mazo@virintel.com (I.M.); 5 Argentys Informatics, LLC, Gaithersburg, MD 20877, USA

**Keywords:** COVID-19, SARS-CoV-2, antibody detection, nucleocapsid, RBD

## Abstract

Since SARS-CoV-2 appeared in late 2019, many studies on the immune response to COVID-19 have been conducted, but the asymptomatic or light symptom cases were somewhat understudied as respective individuals often did not seek medical help. Here, we analyze the production of the IgG antibodies to viral nucleocapsid (N) protein and receptor-binding domain (RBD) of the spike protein and assess the serum neutralization capabilities in a cohort of patients with different levels of disease severity. In half of light or asymptomatic cases the antibodies to the nucleocapsid protein, which serve as the main target in many modern test systems, were not detected. They were detected in all cases of moderate or severe symptoms, and severe lung lesions correlated with respective higher signals. Antibodies to RBD were present in the absolute majority of samples, with levels being sometimes higher in light symptom cases. We thus suggest that the anti-RBD/anti-N antibody ratio may serve as an indicator of the disease severity. Anti-RBD IgG remained detectable after a year or more since the infection, even with a slight tendency to raise over time, and the respective signal correlated with the serum capacity to inhibit the RBD interaction with the ACE-2 receptor.

## 1. Introduction

COVID-19 is a disease caused by a virus from the Coronaviridae family known as the severe acute respiratory syndrome virus 2 (SARS-CoV-2). It is an enveloped virus with a single-stranded positive-sense RNA genome. It was first identified in December 2019 in Wuhan City, China, where a number of patients developed pneumonia symptoms resembling SARS-CoV-1 infection [1]. In March 2020, the WHO officially declared COVID-19 a pandemic.

The incubation period of the COVID-19 infection ranges from 1 to 14 days [2]. The virus is mainly detected in respiratory secretions, and the general transmission of the infection is considered airborne. Immune response is expected to build starting from one week after infection. The levels of IgG antibodies were shown to appear starting from a week after the onset of the disease being detectable in most samples after 20–22 days [3].

SARS-CoV-2 is the seventh known coronavirus to infect people, after 229E, NL63, OC43, HKU1, MERS, and SARS-CoV; while the first four are known as the “common cold” viruses, MERS and SARS-CoV were the cause of severe diseases (with mortality rate of 34% and 9%, respectively [4,5]). All patients infected by SARS-CoV were found to develop IgG antibodies [6], at the same time some patients with mild symptoms of MERS failed to develop detectable levels of IgG specific to MERS-CoV [7]. The human body is known to produce antibodies recognizing multiple epitopes on various proteins in response to a single infection, and, in case of SARS-CoV-2, most of currently available tests measure antibodies to the viral nucleocapsid protein (N), to the spike protein (S), or, specifically, to its receptor-binding domain (RBD). Previous studies on SARS and MERS have shown that IgG specific to S and N have different characteristics in terms of response time, duration, and titers [8,9].

The S protein in its trimeric form is involved in the initial interaction with the receptors of the host cell. This glycoprotein consists of two subunits, S1 and S2, which are separated by the furin protease cleavage site. Cleavage at this site before the entry into the cell occurs only in SARS-CoV-2, not SARS or MERS, and is proposed to be one of the reasons for its higher infectivity [10]. The RBD is represented by amino acids from 333 to 527 of the S1 subunit. It binds the angiotensin converting enzyme 2 (ACE2) receptor on the human host cell surface. The main amino acids involved in this interaction are 438–506 (RBM) [11].

It was shown that the RBD can stimulate production of the antibodies that bind to the virus and prevent it from attaching to the human ACE2 receptors [12,13], thus anti-RBD IgG are often called neutralizing antibodies. In recent studies, however, it was demonstrated that about a third of samples from individuals with previous COVID-19 infection do not seem to contain a detectable number of neutralizing antibodies, and that their low or absent titers positively correlate with the possibility of critical illness and patient death [14]. At the same time, higher concentrations of IgG antibodies to both N and S antigens were observed in patients with severe symptoms than in patients with mild symptoms [15,16]. In another study, about 9% of samples collected from patients with mild symptoms did not show positive results in commercial antibody tests [17], whilst the samples showed no overt signs of immunodeficiency based on peripheral blood cells analysis, and total concentrations of IgG, IgA, and IgM isotypes in serum sampled at the same timepoints were within the normal adult range, indicating no antibody deficiencies. After an additional noncommercial in-house ELISA was performed in that study, low levels of IgG antibodies to RBD were finally detected in these samples. Moreover, all serum samples showed notable neutralizing capabilities in an experiment with serum/virus mixture and confluent Vero cells, indicating that all these patients had mounted a functional humoral immune response against SARS-CoV-2.

Overall, COVID-19 patients with very light symptoms or no symptoms have been studied much less than the individuals with prominent symptoms, mostly due to the fact that they often did not seek medical help, were not hospitalized, and in many cases were not even aware of being sick at the time of infection. In this work, we aimed to analyze the antibody production to both N and RBD antigens and neutralization capabilities of the serum in patients with different levels of disease severity, including individuals with confirmed COVID-19 infection who only experienced anosmia or light fatigue or have not had any kind of symptoms.

## 2. Materials and Methods

### 2.1. Samples

Antibody signal to the N and RBD antigens was measured in the serum of 47 COVID-19 patients. The data were collected from individuals who encountered the infection in the period from March 2020 to February 2021. The sampling and testing were performed from October 2020 to June 2021. Of these 47 samples, 27 cases of infection were confirmed by RT-PCR tests, and the other 20 were previously confirmed by alternative commercial antibody tests. Five samples were obtained after 2019 from individuals who have not been, to their knowledge, infected with SARS-CoV-2. Seven prepandemic negative control samples, tested HIV- and hepatitis-negative, were provided by the Laboratory of cell cultures and cell engineering of the Institute of Cell Biophysics RAS, 89 prepandemic negative samples were obtained from Access Biologicals LLC.

All participants met the inclusion/exclusion criteria. Eligible for this study were individuals of all sexes, aged 12 years and older; healthy or with preexisting medical conditions (other than the SARS-CoV-2 infection) who are considered stable. A stable medical condition is defined as a disease not requiring significant change in therapy or hospitalization during the 3 months before the enrollment. Participants were only considered eligible if they understood and agreed to comply with the study procedures and provided written informed consent.

Additionally, the antibodies signal to the N and RBD antigens was measured for 620 individuals who wished to be tested to find out whether they had been through infection or not.

All procedures were approved by the Commission on Biosafety and Bioethics (Institute of Cell Biophysics—Pushchino Scientific Center for Biological Research of the Russian Academy of Sciences, Permission no. 1 of 12 June 2020) in accordance with Directive 2010/63/EU of the European Parliament.

The patients/participants provided their written informed consent to participate in this study. All necessary patient/participant forms have been signed and archived.

### 2.2. Dual-Antigen Testing ELISA Assay

The signal to anti-RBD and anti-N IgG antibodies was analyzed using the dual-antigen VirIntel assay, previously developed in our laboratory [18]. Each of the 96-well ELISA plates of this testing system was coated with either of the two SARS-CoV-2 antigens diluted in PBS. The N-antigen was produced using a pET-based vector and expressed in a soluble form in an *E. coli* BL21(DE3) strain. The RBD-antigen was expressed from the CMV promoter in HEK293 cells. Each well was coated with either 3.75 μg/mL of RBD-antigen or 4 μg/mL of N-antigen. Each plate had three wells for the threshold identification, where the control antibody solution was added during the analysis.

ELISA was made as described in the assay protocol, with minor modifications. In brief, the wells of the plate, were filled with 98 μL of PBS-T containing 1% casein (1× Casein in PBS ready to use solution, ThermoFisher (Waltham, MA, USA) #37528 with 0.1% TWEEN-20 added). Then, 2 μL of each sample was added to each well, and the plate was incubated for 2 h at 23 °C (RT). After 3 washes with 300 μL of PBS-T, 100 μL of antihuman IgG HRP-conjugated secondary antibody (GenScript (Piscataway, NJ, USA), A01854) diluted 1:3000 in PBS-T + 1% casein was added to the wells. The plate was incubated for 1 h at 23 °C (RT), washed three times with PBS-T and stained with SigmaFast OPD (Sigma (St. Louis, MO, USA), P9187). The resulting absorbance was measured on a Biotek Synergy H1 plate reader (Winooski, VT, USA) at 490 nm. Each sample was assayed in duplicate.

### 2.3. ELISA Result Analysis

The antibody level for each individual was determined by comparison of the obtained optical density value of the respective sample to the threshold of the assay, as specified in the VirIntel test protocol [18]. This threshold value was obtained by measuring the signal for the positive control antibody solution in the respective wells of the assay.

The necessary concentration of this solution was determined during the assay production using a training set of negative (prepandemic) and positive (obtained from individuals with confirmed previous COVID-19 infection) samples, where the signal ranges separating these two cohorts were identified. Then the calibration curves built from a set of antibody dilutions for both N and RBD antigens were used to determine the concentrations that fall within these ranges. The signals from the wells with antibody solutions of these concentrations indicated the lowest possible values that should be considered positive during testing.

The ratio of its own signal to the threshold signal for each sample is referred to as the “signal to cutoff ratio” (S/CO) and the result was considered positive if the S/CO is above 1.

### 2.4. Neutralization Assay

The ability of serum to inhibit the RBD binding to ACE2 receptor was measured using the SARS-CoV-2 Surrogate Virus Neutralization Test (sVNT) Kit (ProteoGenix, Schiltigheim, France) according to the manufacturer’s protocol.

### 2.5. Statistical Analysis

The statistical significance of correlation effects was calculated, and respective *p*-values were obtained using the Pearson’s correlation coefficient method. Fisher’s exact test was used to compare categorical variables. *p*-value of <0.05 indicated statistical significance. To determine the 95% confidence interval (95% CI) for the result assessment regarding the exact binomial, Bayesian credible interval (Jeffrey’s interval) [19] method was used.

## 3. Results

Using the VirIntel Dual-Antigen assay [18], we measured the IgG antibodies to the N and RBD antigens in the serum of 47 COVID-19 patients. All except one demonstrated positive signal for antibodies to the RBD antigen, for the N antigen, a positive signal was observed for 34 samples.

**Figure 1:**
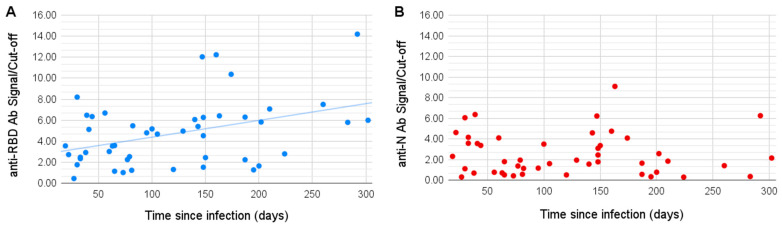
Correlation of time since infection (TSI) with the antibody signal. The S/CO to the anti-RBD (**A**) and anti-N (**B**) antibodies are shown on the vertical axes, TSI is shown on the horizontal axes. The signal to anti-RBD antibodies shows a positive correlation with TSI (R 0.38).

Surprisingly, we observed a slight positive correlation between the time since infection (TSI, number of days between the onset of the disease and the sample being taken) and the RBD antibody signal (Pearson coefficient of 0.38, *p*-value 0.008). Four of the patients with TSI over 20 weeks held the record values for the antibodies to the RBD antigen, exceeding the threshold level 10–14 times. The signal of antibodies to the N antigen, on the contrary, showed no significant correlation with TSI (Figure 1).

**Figure 2:**
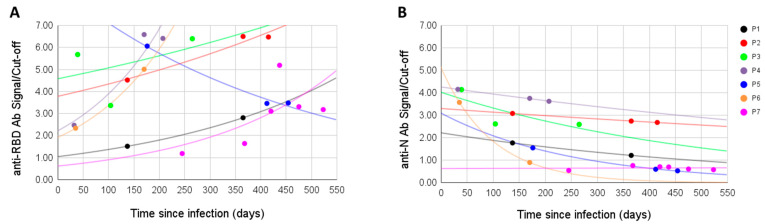
Antibodies to the RBD antigen (**A**) and to the N antigen (**B**) measured at different time points for seven patients (P1–P7).

For seven patients, we measured the antibody signal at different time points. Antibodies to the RBD antigen did not decline with time, on the contrary, the signal was often higher after the initial sampling (in four cases it raised, in one lowered, in one raised then lowered, and in one lowered then raised). Overall, the anti-RBD signal was positive in all samples, even when sampling was performed over a year after the infection (Figure 2A). On the other hand, in all six initially positive anti-N samples the signal dropped with time, in one case going from positive to negative; the seventh sample was anti-N negative from the first sampling and remained so (Figure 2B).

**Figure 3:**
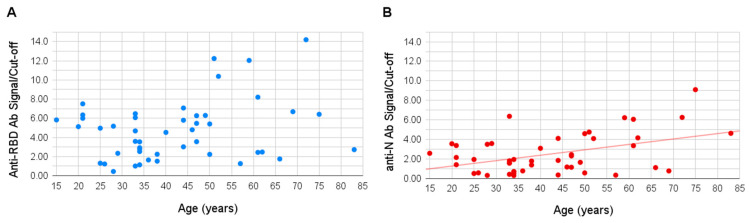
Correlation of patient age with antibody signal. The S/CO to the anti-RBD (**A**) and anti-N (**B**) antibodies are shown on the vertical axes, and the patient’s age is shown on the horizontal axes. The S/CO to anti-N antibodies shows a positive correlation with age (R 0.49).

Age of the sampled individuals varied between 15 and 83 years. No significant correlation was observed between the patient’s age and signal of the antibodies to the RBD antigen, however, a slight positive correlation was shown for the antibodies to the N antigen (Pearson coefficient of 0.49, *p*-value 0.003), the signal to which appeared higher in older people (Figure 3).

**Figure 4:**
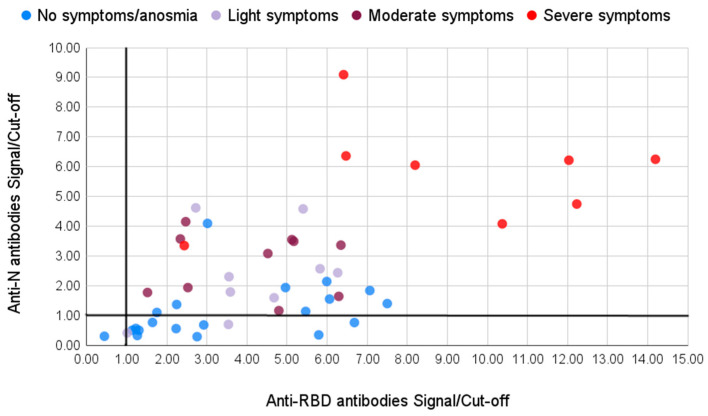
Symptom correlation with anti-N and anti-RBD IgG antibody signals. Black lines highlight cutoff value of 1 for each axis, dots higher (for the anti-N) or to the right (for the anti-RBD) are considered positive. In total, 13 out of 26 of the asymptomatic (blue) and light symptom (light purple) cases had S/CO to the anti-N lower than 1 and were thus considered negative.

We divided the samples into two groups based on the symptoms the patients had during the infection. The first group consisted of 29 patients who had no or light symptoms: 20 of them did not experience any symptoms at all or only had light fatigue and anosmia (marked blue in Figure 4); another nine patients had light fever for one or a few days, some of them also had light cough and anosmia (marked light purple in Figure 4). The second group consisted of 18 patients who had moderate (marked violet in Figure 4) or severe (marked red in Figure 4) symptoms of the disease, with high fever lasting for several days, cough, headaches, and pneumonia, and four cases required hospitalization.

All of the samples except one demonstrated an above-threshold level of antibodies to the RBD antigen (Table 1). This person only experienced mild fatigue during the course of the infection. Among the group with moderate/severe symptoms all the samples demonstrated a positive result for the N antigen. In the group with light symptoms, however, among 26 samples 13 showed negative results for the anti-N IgG. Thus, IgG antibodies to the N antigen are not detected in half of the cases in individuals with light/no symptoms, showing a significant trend (*p*-value 0.0001).

This trend was also reflected in a larger scale general public testing (Table 1). Among 620 individuals from general public testing, 66 (10.6%) demonstrated above-threshold levels for both antigens and the results were presented to them as positive. Another 554 individuals had one or both antigen levels below the required threshold. Of these, 100 were positive only for the RBD antibodies, and 22 were positive only for the N antibodies. Among the 96 prepandemic negative controls two tested positive for the N antigen, and one tested positive for the RBD antigen.

**Figure 5:**
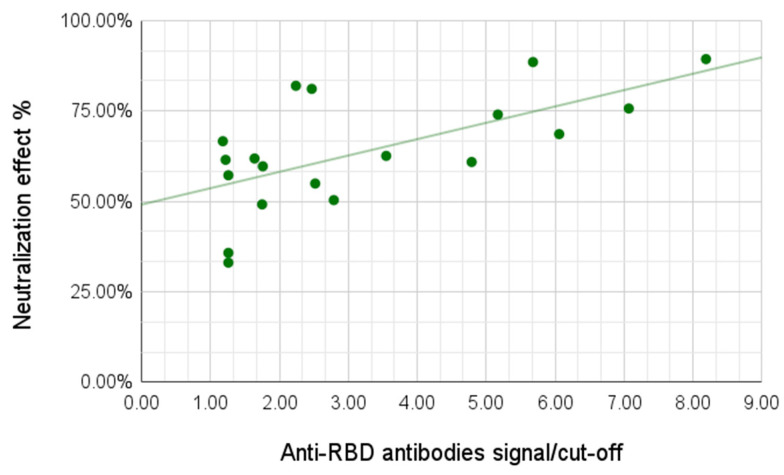
Correlation of anti-RBD antibody signal with ACE-2 neutralization effect (R 0.59).

We then observed the neutralization capabilities of the serum samples using a surrogate neutralization system assessing the ability of antibodies to prevent binding of the viral antigen to the human ACE-2 receptor. For this analysis we tested 24 samples, including 13 samples from the group of individuals with light/no symptoms and all 11 samples from the group with moderate/severe symptoms. The neutralization effect varied between 33.06% and 89.31%, with the average for the group with no/light symptoms of 54.89% and for the group with moderate/severe symptoms of 73.99%. A correlation between antibody signal and neutralization effect was demonstrated; a higher signal for the anti-RBD antibodies yielded higher neutralization effect (Pearson coefficient 0.59, *p*-value 0.002) (Figure 5).

Seven negative controls from the prepandemic era demonstrated no neutralizing capabilities. Surprisingly, all five negative controls from after 2019 obtained from people who have not been infected with SARS-CoV-2 to their knowledge and demonstrated no detectable anti-RBD or anti-N IgG antibodies showed significant neutralizing effect (between 55% and 65%).

## 4. Discussion

Within the scope of our study, half of the samples from patients with light or asymptomatic course of the COVID-19, while having a detectable level of anti-RBD antibodies, did not demonstrate positive antibody signal to the nucleocapsid protein of SARS-CoV-2. Our findings bring up the question of whether measuring antibodies to the nucleocapsid protein is a valid method for determining whether a person has been through the infection or not.

One of the possible explanations to this phenomenon is that the immune response is faster and more efficient in the patients with light course of the disease; when the virus enters the body the antibodies to the spike protein, and, specifically, to RBD, are developed quickly, almost entirely blocking viral entry into the host cells. The reproduction of the virus would mostly be halted, and there would be a lack of exposure of other viral proteins to the immune system, which normally happens during the course of severe infection with many cycles of viral reproduction [20,21,22]. The leukocytes would not present these proteins, including the nucleocapsid protein, as antigens, and the respective antibodies would not be produced by the B-lymphocytes.

From 620 routinely tested samples with unknown background, 66 (10.6%) demonstrated positive results for both anti-N and anti-N IgG. Among the rest, 22 individuals were positive only for the anti-N antibodies, and 100 were positive only for the anti-RBD antibodies. During testing we expect a certain rate of false positive results. The assay has specificity for anti-N antibody detection of 97.8%, originally validated on 89 negative prepandemic samples [18]. In the current large cohort, 22 cases of candidate false positives out of 620 would indicate the specificity of 96.4%, which is not a significant change from the original value. On the other hand, specificity of the assay to the anti-RBD antibodies is 98.9%, and the observation of possible 100 false positive results in this large cohort would bring it down to 83.9%, which is much less plausible.

The same reasoning can be applied to the false negative rates. Possible 22 false negative results change the original sensitivity of the assay regarding the RBD antigen from 100% to 96.4%, which does not seem drastic, but possible 100 false negative results for the N antigen change the respective sensitivity from 93.6% to 83.9%, which is more significant, and, again, less plausible. Thus, some of the individuals that tested positive for anti-RBD antibodies, but negative for anti-N, have likely encountered the infection in the past.

This trend in a cohort from the general public further strengthens the idea that while anti-RBD antibodies are produced in most cases after the infection, the antibodies to the N antigen are not always detectable. It is also important that the original set of the positive samples for the assay validation was collected at the beginning of the pandemic from individuals admitted to hospitals; these patients were likely to have severe symptoms of the disease. Some individuals from a larger cohort who wished to be tested for antibodies, however, had light symptoms or no symptoms at all. Based on these observations, it seems best to redefine the significance of measuring the level of the anti-N antibodies in overall COVID-19 testing.

The studies on the tendency for the antibody levels to drop over time have produced somewhat contradictory results. For example, a decline in the anti-RBD IgG antibodies has been reported for individuals with a mild course of the disease (the levels decreased by about half every 73 days) [23]. At the same time, other recent studies have shown that IgGs against the spike protein remain detectable even after 11 months postinfection, seemingly maintained by long-lived bone marrow plasma cells (BMPCs) [24]. Within the scope of our study, we observed a slight positive correlation between the signal to anti-RBD antibodies and the time between the onset of infection and sampling. This effect may be due to the limited size of the studied cohort; nonetheless, the RBD antibody signal was surprisingly high in a significant number of patients who were infected a long time ago. Four of the patients with TSI over 20 weeks demonstrated the highest signals among the whole study. However, we see that the levels of anti-N antibodies, being higher than anti-RBD immediately after the infection, showed a clear decline with time in a cohort of patients with several samplings taken at different time points. In some patients with a severe course of the disease, anti-N IgG signals were still high after 10–12 months postinfection, but in the cases with mild symptoms they disappeared much faster.

Overall, according to our observations, the initial anti-RBD/anti-N ratio may be an indicator of the disease severity; lung lesions, for example, always resulted in high anti-N Ab, lowering this ratio. In asymptomatic patients or patients with anosmia as a sole symptom, the levels of anti-RBD IgG were comparable or higher than that in patients with severe symptoms, while anti-N IgG were much lower, in half cases negative. Thus, a higher anti-RBD/anti-N ratio possibly reflects less severe symptoms.

No significant correlation was observed between the patient’s age and production of the anti-RBD antibodies, possibly, again, due to the limitations of the cohort size. However, a positive correlation was found for the anti-N antibodies; the signal was, on average, higher in older people. This finding seems to be in agreement with several previous studies; in particular, one of them showed that patients aged 19 to 24 years had significantly lower IgG levels than the adults aged 51 to 80 years [25]; another compared patients aged 15 to 39 years to older groups and also showed that younger individuals produced lower levels of IgGs [26].

A positive correlation between the anti-RBD antibody signal and the serum neutralization capabilities was demonstrated, a higher signal for the anti-RBD antibodies yielded higher neutralization effect. The difference in neutralization effects between the group with no/light symptoms and the group with moderate/severe symptoms was significant, the latter showing higher neutralization capabilities, in agreement with previous studies [27].

A surprising finding was the neutralization effect in negative controls. We have studied seven negative controls from the prepandemic era and five negative controls from 2020–2021 collected from the individuals who have not been infected with SARS-CoV-2 to their knowledge. No antibodies to either N or RBD antigens were detected in those samples. While none of the prepandemic serum samples demonstrated neutralizing capabilities, all five of the presumably negative pandemic era serum samples showed a significant neutralizing effect.

The latter individuals could have been previously unknowingly infected with SARS-CoV2, and had a completely asymptomatic course of the disease. However, none of the antibody tests, including commercially available Abbott Architect Quant II and DiaSorin, detected IgG antibodies to either RBD or N antigens in the respective sera. The neutralization effect could have been mediated by other classes of antibodies, such as IgM or IgA, although the lack of IgG antibodies in this scenario is difficult to explain, since in all cases of the confirmed COVID-19 infections anti-RBD IgG antibodies were registered. False negative results are possible, but extremely unlikely to be observed for five cases out of five.

Another group [28] observed a similar effect of the immune response from individuals without knowledge of a previous SARS-CoV2 infection. The authors state that healthy donors examined during the pandemic exhibited increased numbers of SARS-CoV-2-specific T cells, but showed no humoral response. As an explanation, they also suggest probable exposure to the virus, which resulted in either asymptomatic infection without antibody secretion or activation of preexisting immunity.

Obtaining a sufficient number of samples from individuals with very little to no symptoms but confirmed SARS-CoV-2 infection was an important challenge of this work that we succeeded to overcome. Despite the limitations related to the size of the studied cohort, we observed a significant number of post-infection cases from individuals who had not seem to develop anti-nucleocapsid IgG antibodies; all of them were light or asymptomatic cases. We also show that while the humoral response of the patients proved to be largely based on the individual characteristics of the immune system, the anti-RBD IgG antibodies were detectable in the serum even after a year or more after the infection. Simultaneous measurement of the antibody signal to both N-protein and RBD of S1 using our unified dual-antigen assay significantly lowered possible bias appearing from using and comparing results from different testing systems.

We suggest that the anti-RBD/anti-N IgG antibody ratio may serve as an indicator of the disease severity. Importantly, current testing systems in diagnostics aimed solely at the anti-N antibody detection may not always be sufficient in reporting whether a person had encountered the infection in the past.

## Figures and Tables

**Table 1:** Distribution of RBD-positive and N-positive signals in different sets of samples: negative controls, confirmed COVID-19 cases, and general public cohort. The total number of samples tested in each category is shown in the table heading. The percentage of positive samples for each serotype is presented with 95% confidence interval shown in parentheses.

Detected Ab	Prepandemic (96)	Confirmed COVID-19 Cases (47)	Unknown COVID-19 Status (620)
RBD+/N+	0% (0–2.58%)	72.34% (58.50–83.52%)	10.65% (8.40–13.26%)
RBD+/N−	1.12% (0.04–5.13%)	25.53% (14.78–39.19%)	16.13% (13.39–19.18%)
RBD−/N+	2.25% (0.4–6.5%)	0% (0–5.18%)	3.55% (2.30–5.33%)
RBD−/N−	96.63% (93.49–99.56%)	2.13% (0.23–9.52%)	66.13% (62.34–69.77%)

## Data Availability

All data generated and analyzed during this study are included in this published article. Raw data supporting the findings of this study are available from the corresponding author on request. Data regarding patients presented in this study are not publicly available due to ethical reasons, e.g., containing information that could compromise the privacy of research participants.

## References

[1] Zhou P., Yang X.-L., Wang X.-G., Hu B., Zhang L., Zhang W., Si H.-R., Zhu Y., Li B., Huang C.-L. (2020). A pneumonia outbreak associated with a new coronavirus of probable bat origin. Nature.

[2] Elias C., Sekri A., Leblanc P., Cucherat M., Vanhems P. (2021). The incubation period of COVID-19: A meta-analysis. Int. J. Infect. Dis..

[3] Li K., Huang B., Wu M., Zhong A., Li L., Cai Y., Wang Z., Wu L., Zhu M., Li J. (2020). Dynamic changes in anti-SARS-CoV-2 antibodies during SARS-CoV-2 infection and recovery from COVID-19. Nat. Commun..

[4] Zumla A., Hui D.S., Perlman S. (2015). Middle East respiratory syndrome. Lancet.

[5] Sorensen M.D., Sørensen B., Gonzalez-Dosal R., Melchjorsen C.J., Weibel J., Wang J., Jun C.W., Huanming Y., Kristensen P. (2006). Severe Acute Respiratory Syndrome (SARS): Development of Diagnostics and Antivirals. Ann. N. Y. Acad. Sci..

[6] Liu W., Fontanet A., Zhang P., Zhan L., Xin Z., Baril L., Tang F., Lv H., Cao W. (2006). Two-Year Prospective Study of the Humoral Immune Response of Patients with Severe Acute Respiratory Syndrome. J. Infect. Dis..

[7] Alshukairi A.N., Khalid I., Ahmed W.A., Dada A.M., Bayumi D.T., Malic L.S., Althawadi S., Ignacio K., AlSalmi H.S., Al-Abdely H.M. (2016). Antibody Response and Disease Severity in Healthcare Worker MERS Survivors. Emerg. Infect. Dis..

[8] Zhao J., Wang W., Wang W., Zhao Z., Zhang Y., Lv P., Ren F., Gao X.-M. (2007). Comparison of Immunoglobulin G Responses to the Spike and Nucleocapsid Proteins of Severe Acute Respiratory Syndrome (SARS) Coronavirus in Patients with SARS. Clin. Vaccine Immunol..

[9] Leung D.T.M., Tam F.C.H., Ma C.H., Chan P., Cheung J.L.K., Niu H., Tam J.S.L., Lim P.L. (2004). Antibody Response of Patients with Severe Acute Respiratory Syndrome (SARS) Targets the Viral Nucleocapsid. J. Infect. Dis..

[10] Örd M., Faustova I., Loog M. (2020). The sequence at Spike S1/S2 site enables cleavage by furin and phospho-regulation in SARS-CoV2 but not in SARS-CoV1 or MERS-CoV. Sci. Rep..

[11] Lan J., Ge J., Yu J., Shan S., Zhou H., Fan S., Zhang Q., Shi X., Wang Q., Zhang L. (2020). Structure of the SARS-CoV-2 spike receptor-binding domain bound to the ACE2 receptor. Nature.

[12] Rogers T.F., Zhao F., Huang D., Beutler N., Burns A., He W.-T., Limbo O., Smith C., Song G., Woehl J. (2020). Isolation of potent SARS-CoV-2 neutralizing antibodies and protection from disease in a small animal model. Science.

[13] Zost S.J., Gilchuk P., Case J.B., Binshtein E., Chen R.E., Nkolola J.P., Schäfer A., Reidy J.X., Trivette A., Nargi R.S. (2020). Potently neutralizing and protective human antibodies against SARS-CoV-2. Nature.

[14] Dispinseri S., Secchi M., Pirillo M.F., Tolazzi M., Borghi M., Brigatti C., De Angelis M.L., Baratella M., Bazzigaluppi E., Venturi G. (2021). Neutralizing antibody responses to SARS-CoV-2 in symptomatic COVID-19 is persistent and critical for survival. Nat. Commun..

[15] Long Q.-X., Tang X.-J., Shi Q.-L., Li Q., Deng H.-J., Yuan J., Hu J.-L., Xu W., Zhang Y., Lv F.-J. (2020). Clinical and immunological assessment of asymptomatic SARS-CoV-2 infections. Nat. Med..

[16] Liu Z.-L., Liu Y., Wan L.-G., Xiang T.-X., Le A.-P., Liu P., Peiris M., Poon L.L.M., Zhang W. (2020). Antibody Profiles in Mild and Severe Cases of COVID-19. Clin. Chem..

[17] Marklund E., Leach S., Axelsson H., Nyström K., Norder H., Bemark M., Angeletti D., Lundgren A., Nilsson S., Andersson L.-M. (2020). Serum-IgG responses to SARS-CoV-2 after mild and severe COVID-19 infection and analysis of IgG non-responders. PLoS ONE.

[18] Komarov A., Kaznadzey A., Li Y., Kireeva M., Mazo I. (2021). Dual-Antigen System Allows Elimination of False Positive Results in COVID-19 Serological Testing. Diagnostics.

[19] Brown L.D., Cai T.T., DasGupta A. (2001). Interval Estimation for a Binomial Proportion. Stat. Sci..

[20] Abbas A.K., Lichtman A.H., Pillai S. (2019). Basic Immunology E-Book: Functions and Disorders of the Immune System.

[21] Murin C.D., Wilson I.A., Ward A.B. (2019). Antibody responses to viral infections: A structural perspective across three different enveloped viruses. Nat. Microbiol..

[22] Slifka M.K. (2004). Immunological memory to viral infection: Commentary. Curr. Opin. Immunol..

[23] Ibarrondo F.J., Fulcher J.A., Goodman-Meza D., Elliott J., Hofmann C., Hausner M.A., Ferbas K.G., Tobin N.H., Aldrovandi G.M., Yang O.O. (2020). Rapid Decay of Anti–SARS-CoV-2 Antibodies in Persons with Mild Covid-19. N. Engl. J. Med..

[24] Turner J.S., Kim W., Kalaidina E., Goss C.W., Rauseo A.M., Schmitz A.J., Hansen L., Haile A., Klebert M.K., Pusic I. (2021). SARS-CoV-2 infection induces long-lived bone marrow plasma cells in humans. Nature.

[25] Yang H.S., Costa V., Racine-Brzostek S.E., Acker K.P., Yee J., Chen Z., Karbaschi M., Zuk R., Rand S., Sukhu A. (2021). Association of Age with SARS-CoV-2 Antibody Response. JAMA Netw. Open.

[26] Wu F., Liu M., Wang A., Lu L., Wang Q., Gu C., Chen J., Wu Y., Xia S., Ling Y. (2020). Evaluating the Association of Clinical Characteristics With Neutralizing Antibody Levels in Patients Who Have Recovered From Mild COVID-19 in Shanghai, China. JAMA Intern. Med..

[27] Putcharoen O., Wacharapluesadee S., Ni Chia W., Paitoonpong L., Tan C.W., Suwanpimolkul G., Jantarabenjakul W., Ruchisrisarod C., Wanthong P., Sophonphan J. (2021). Early detection of neutralizing antibodies against SARS-CoV-2 in COVID-19 patients in Thailand. PLoS ONE.

[28] Shomuradova A.S., Vagida M.S., Sheetikov S.A., Zornikova K.V., Kiryukhin D., Titov A., Peshkova I.O., Khmelevskaya A., Dianov D.V., Malasheva M. (2020). SARS-CoV-2 Epitopes Are Recognized by a Public and Diverse Repertoire of Human T Cell Receptors. Immunity.

